# Supplementary material for: NetTCR-2.0 enables accurate prediction of TCR-peptide binding by using paired TCRα and β sequence data
Source: Commun Biol. 2021 Sep 10;4:1060. doi: 10.1038/s42003-021-02610-3 (PMC8433451; doi:10.1038/s42003-021-02610-3)
Supplement: Supplementary file 2 — Supplementary Information [file 42003_2021_2610_MOESM2_ESM.pdf]

Supplementary Information for

"NetTCR-2.0 enables accurate prediction of TCR-peptide binding by using paired TCRα and β sequence data"

Alessandro Montemurro, Viktoria Schuster, Helle Rus Povlsen, Amalie Kai Bentzen, Vanessa Jurtz, William D. Chronister, Austin Crinklaw, Sine R. Hadrup, Ole Winther, Bjoern Peters, Leon Eyrich Jessen, and Morten Nielsen[$]

[$] Corresponding author: morni@dtu.dk

Supplementary Note 1

pHMM based k-mer method for CDR3β loop sequence excision

As the IEDB is based on collecting published sequence data, the raw CDR3β data downloaded from the IEDB contained not only the CDR3 segment of the VDJ-recombination, but also in some cases included flanking parts of various lengths spanning into the V- and J-segments. To remove these potential excess flanking residues, a profile Hidden Markov Model (pHMM) k-mer based scoring method was developed to extract the correct CDR3β-sequence. Here, the sequenced CDR3β repertoire from 20 healthy donors included in Savola et al.[S1] was used. This data set consists of a total of 487,787 CDR3 sequences of which 405,588 are non-NA, 398,139 of these contain only the 20 standard proteogenic amino acids. A further 352,116 of these are unique and of these 348,249 matched the canonical CDR3 motif “Cxxx...xxx[FW]”. Removing the c-terminal “C” and the N-terminal “[FW]”, resulted in an average length of 12.7 with a standard deviation of 1.8. The final data set was then created by randomly selecting 100,000 sequences to be used for training, leaving the remaining 248,249 for evaluation. Using this setup, a profile Hidden Markov Model was trained using the Baum–Welch algorithm implemented in the Aphid package[S2]. The resulting pHMM model was then used to score each of the 248,249 evaluation sequences using the Viterbi algorithm, resulting in a k-dependent score distribution (Supplementary Figure 9a) reflecting the underlying CDR3β length distribution (Supplementary Figure 9b). Next, the raw IEDB data containing a total of 25,300 CDR3β-pMHC data points of which 13,274 were specific for HLA-A*02:01, further subsetting to 9-mer peptides, yielded 12,353 data points. Removing CDR3β-sequences containing non-standard 1-letter amino acid symbols resulted in 12,223 data points and finally non-trimmed CDR3β-sequences were required to have a length of at least 5, yielding a final data set of 12,222 data points. As a first step, 3,400 CDR3β-sequences with a N-terminus “C” and a C-terminus “F” or “W” were stripped of said flanks. The remaining 8,822 sequences were digested into all possible nested k-mers (5 <= k <= CDR3β-length) and Viterbi-scored using the trained pHMM. The best scoring k-mer was recorded. Finally, in case the k-mer had a higher Viterbi-score than that of the original full length CDR3β, the trimming was accepted replacing the original CDR3β-sequence. This procedure resulted in trimming 911 sequences corresponding to ~7.5% of the 12,222 sequences in the IEDB CDR3β data set. Selecting the unique CDR3β-peptide pairs from this trimmed data resulted in 11,845 data points and removing promiscuous TCRs yielded 11,122 unique CDR3β-sequences and 163 unique peptides. Finally, requiring CDR3β-sequences to have a length of at least 8 amino acids 18 at most (corresponding to 99% of TCRs in the Savola

et al. data set (Supplementary Figure 9b)), resulted in 10,987 CDR3β-sequences covering 163 peptides.

Supplementary References

S1. Savola, P. *et al.* Somatic mutations in clonally expanded cytotoxic T lymphocytes in patients with newly diagnosed rheumatoid arthritis. *Nat. Commun.* **8**, 15869 (2017).

S2. Wilkinson, S. P. aphid: an R package for analysis with profile hidden Markov models. *Bioinformatics* **35**, 3829–3830 (2019).

Supplementary Figures

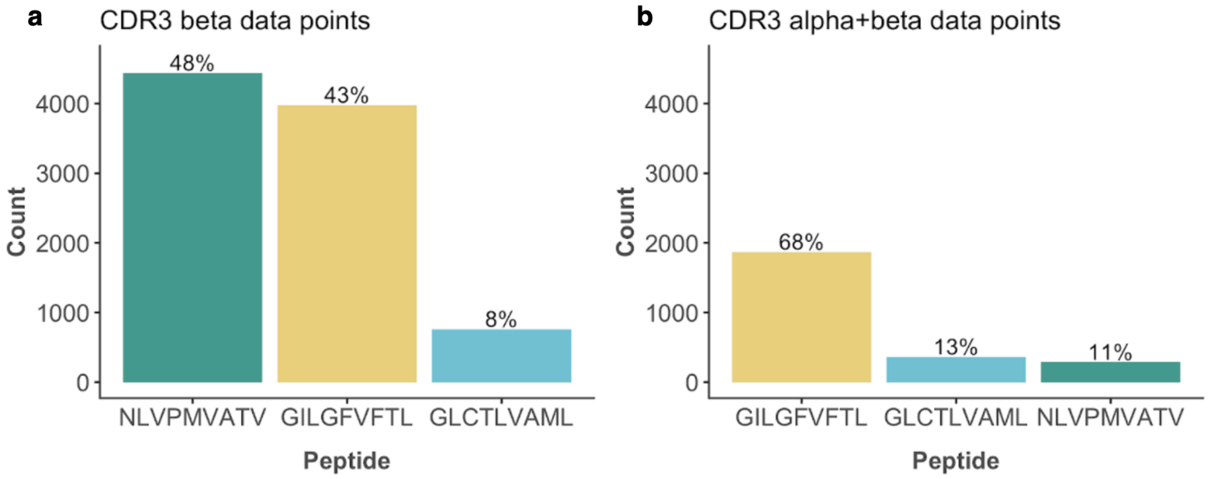

Supplementary Figure 1. Counts of unique data points per peptide. Count for the data sets consisting of only CDR3 β chains (**a**) and both CDR3 α and β chains (**b**). The percentages above bars indicate the representation of peptides in the data.

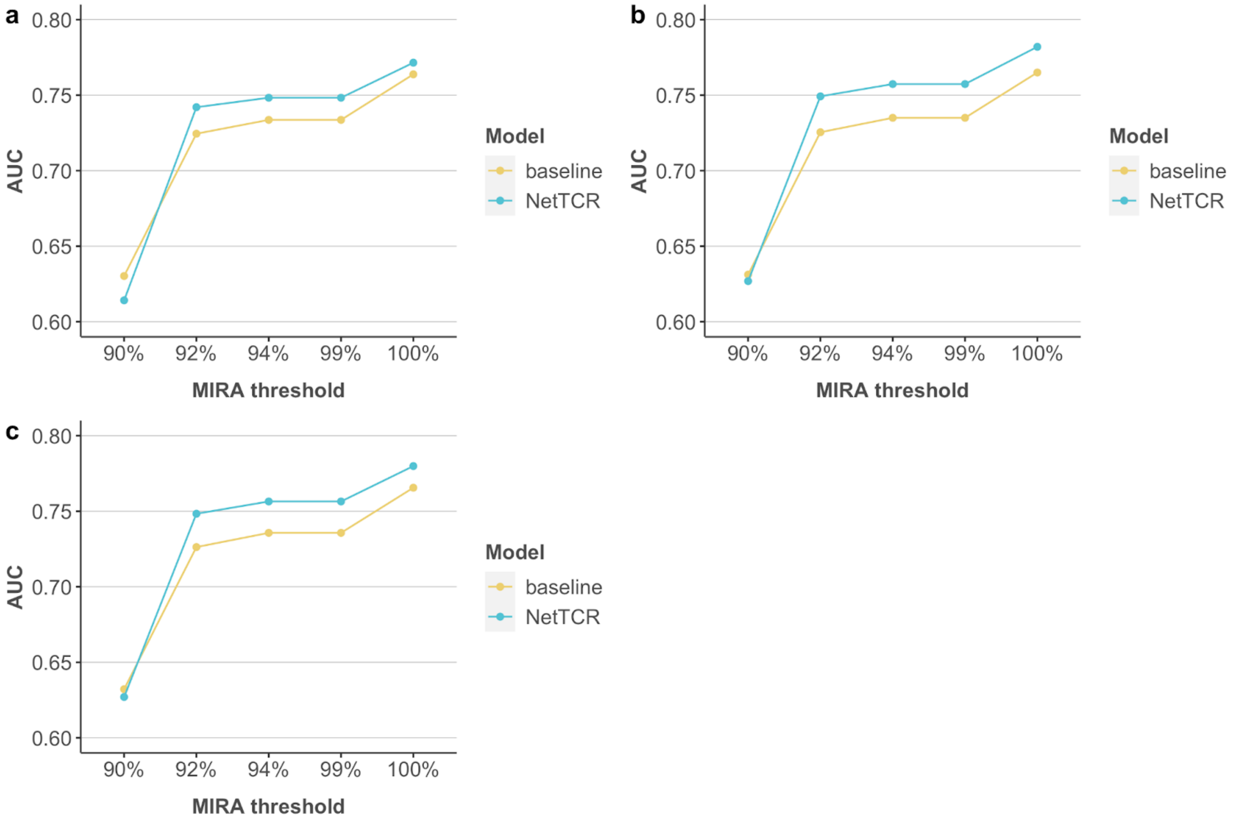

Supplementary Figure 2. Overall AUCs of the CDR3 beta models on the external evaluation MIRA data at different redundancy thresholds of the models trained on the (**a**) 90%, (**b**) 92% and (**c**) 99% partitioned training set.

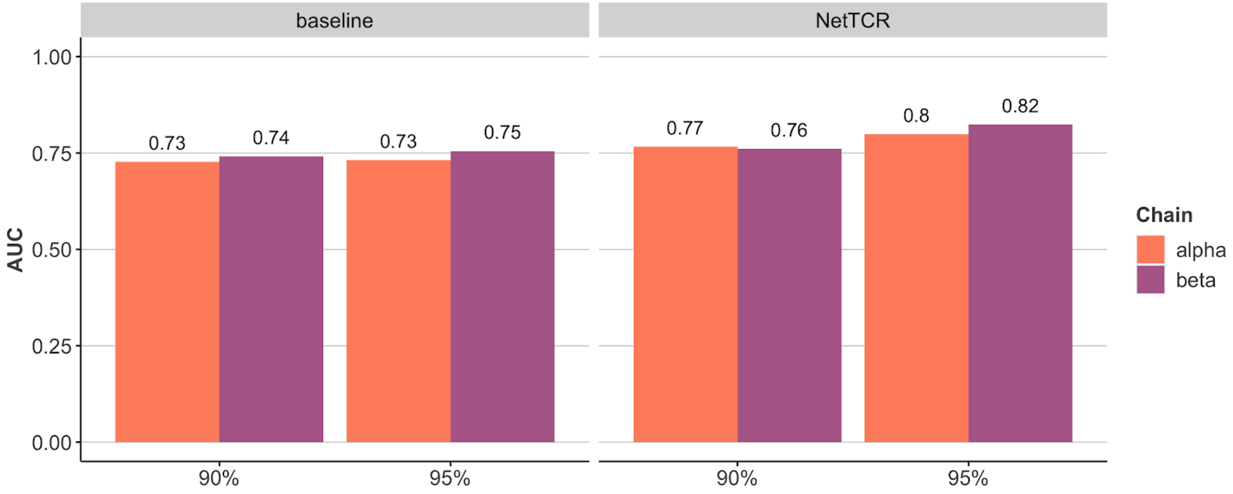

Supplementary Figure 3. Performance of models trained on single-chain data. Overall AUCs evaluated via cross-validation for the different partitioning thresholds. The single-chain data sets were partitioned using a chain-specific partitioning approach.

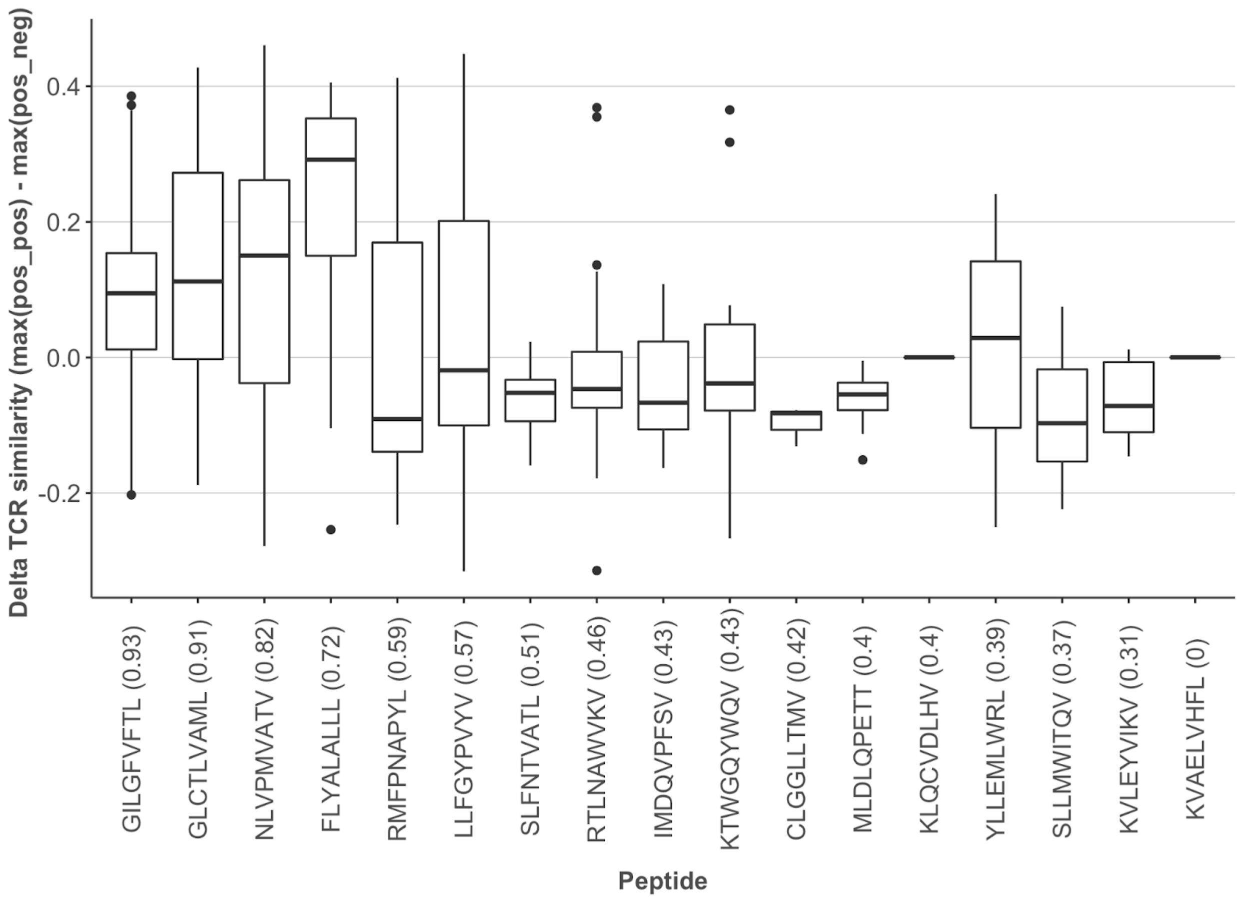

Supplementary Figure 4. Correlation between the performance of the paired chain NetTCR model and the difference between positive and negative data points. The x-axis presents peptides sorted by AUC of the paired chain NetTCR model from 95% partitioning (AUC values indicated next to the peptide). The boxplot shows differences in similarity per peptide between different partitions (see text).

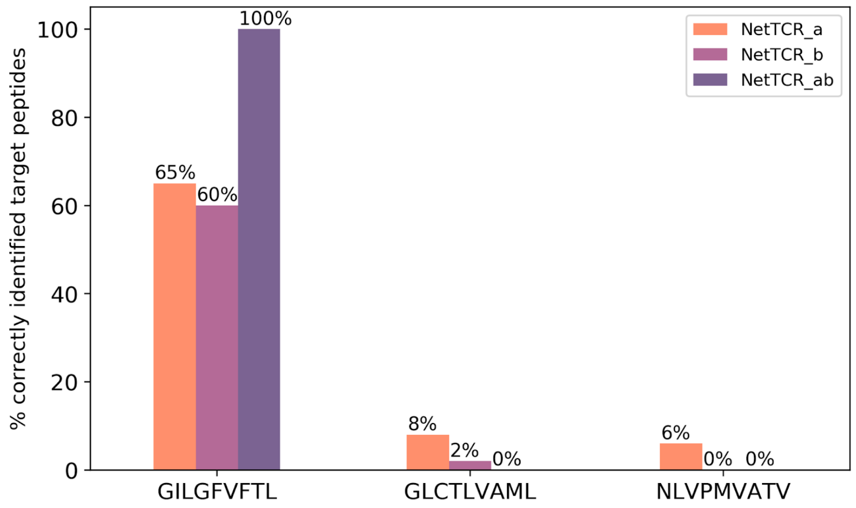

Supplementary Figure 5. Peptide ranking analysis for the paired-chains model trained with a wrong TCR-peptide combination. The TCRs in the training set were paired with a wrong peptide and a model was trained on the mismatched dataset. After, each TCR positive to GIL, GLC, or NLV peptide was paired to the other two peptides and a binding prediction was obtained. The percentages show the proportion of TCRs for which the predicted lowest-ranking peptide matched with the "true" target peptide.

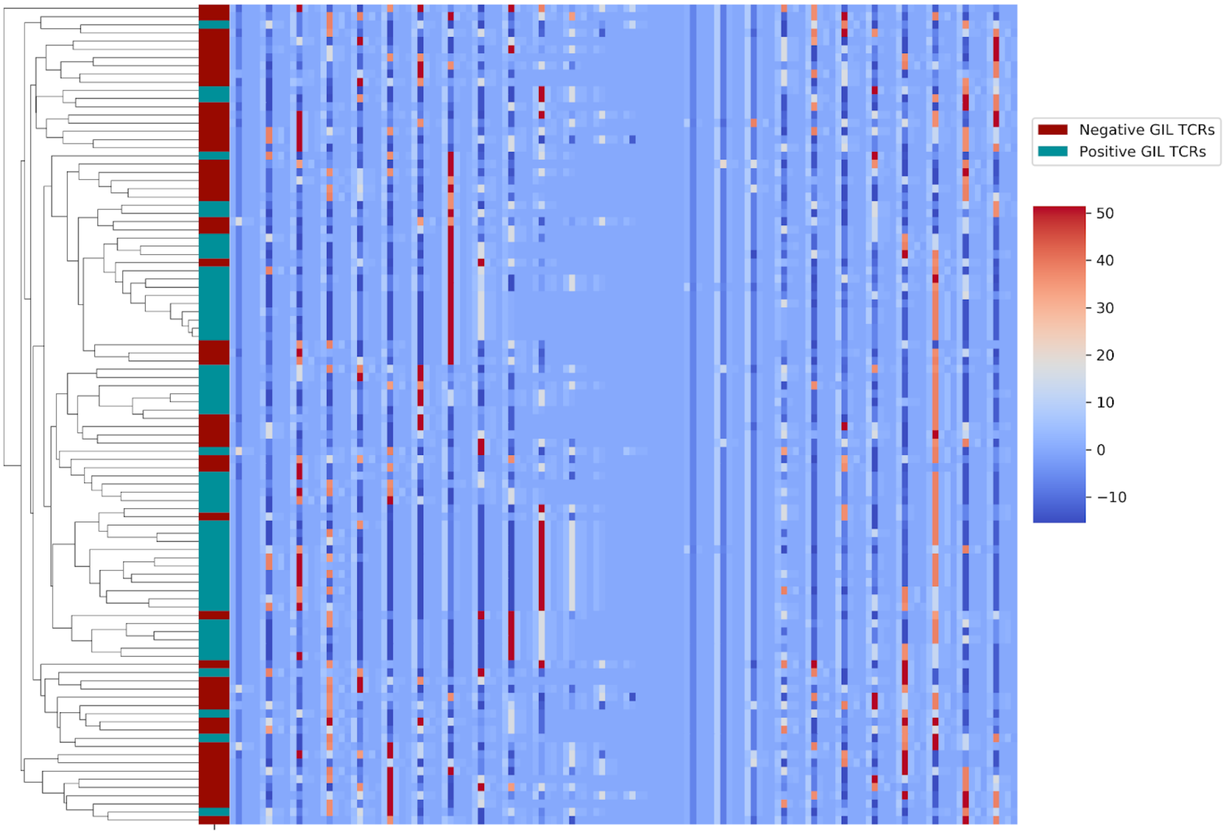

Supplementary Figure 6. Hierarchically-clustered heatmap of a random set of 50 positive and 50 negative TCRs GIL TCRs encoded using the physico-chemical features.

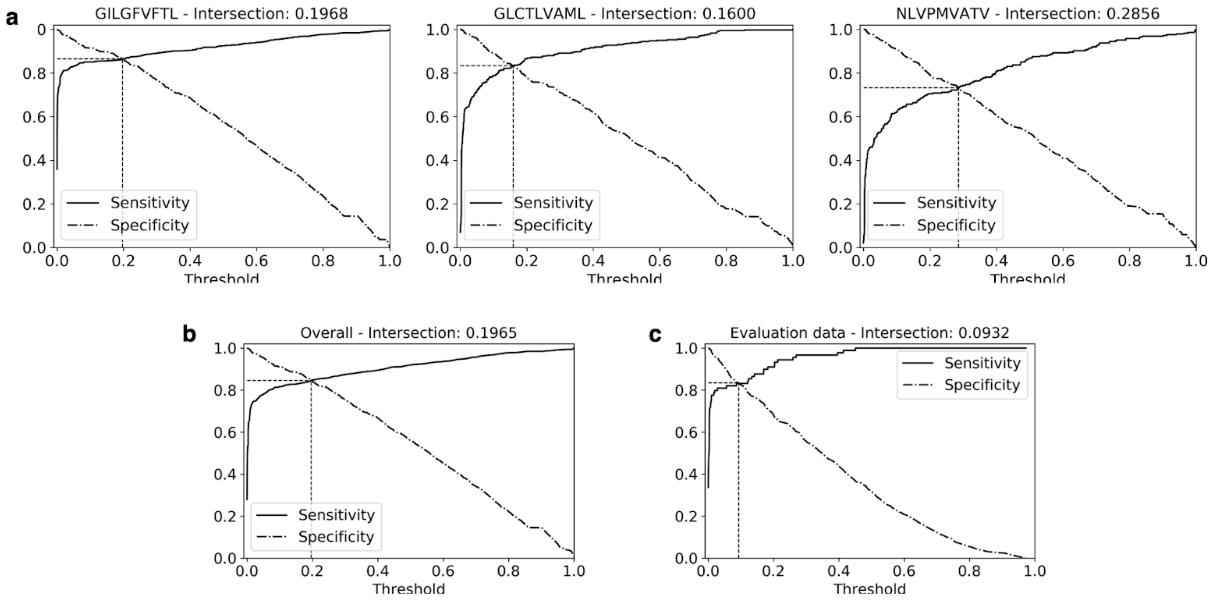

Supplementary Figure 7. Sensitivity and specificity curves as a function of the decision thresholds for NetTCR_αβ. The curves were plotted using peptide-specific percentile rank scores (**a**), all the percentile scores from cross-validation (**b**), and the scores from the external evaluation predictions (**c**).

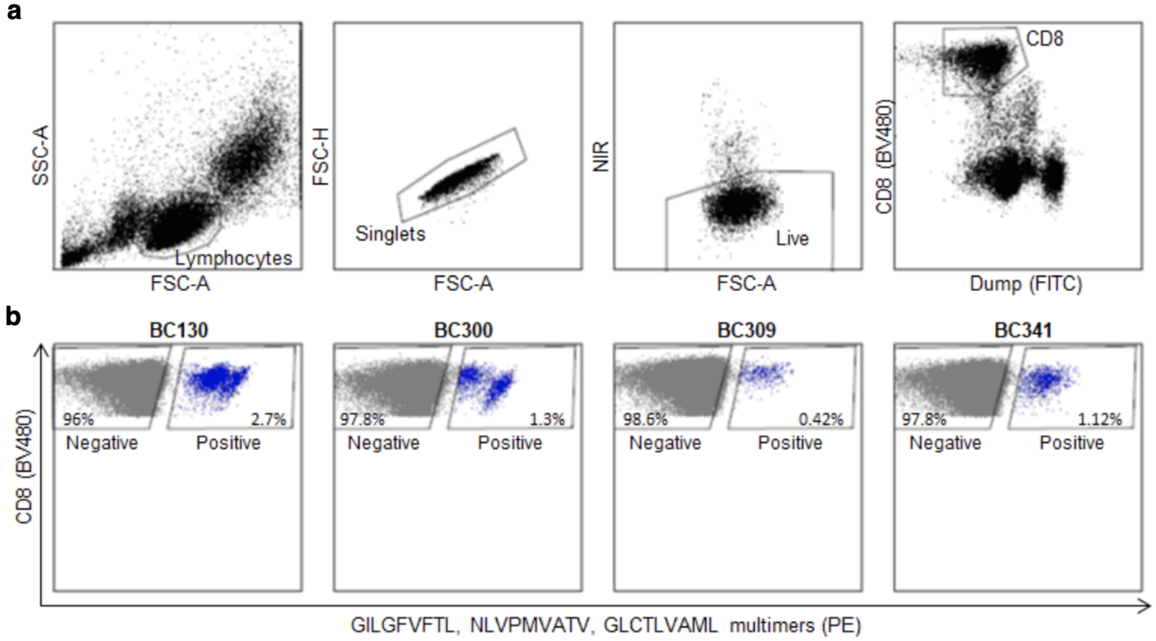

Supplementary Figure 8. Gating strategy and sorted populations used for generating the novel independent paired TCR dataset. (a) Shows an example of the initial gating of CD8+ T cells (BC341). **(b)** Shows the sorted positive and negative populations of total CD8+ T cells from all four samples included.

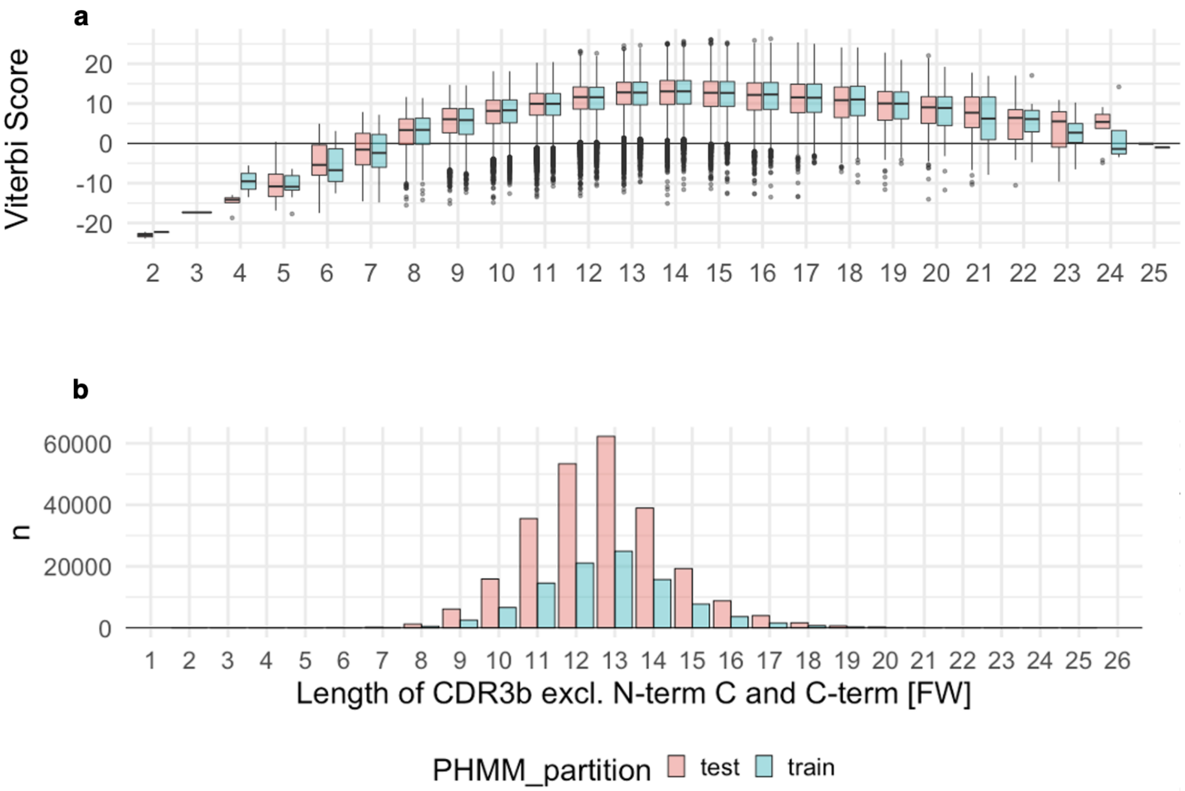

Supplementary Figure 9. (**a**) K-dependent distribution of pHMM derived Viterbi scores. (**b**) The length distribution of CDR3β-sequences. All stratified on the pHMM-test/training partition.

	**Predetermined responses**			**# of sorted cells**	
	**GILGFVFTL**	**GLCTLVAML**	**NLVPMVATV**	**Positive subset**	**Negative subset**
**BC130**			2.1%	4698	75000
**BC300**	0.7%	0.9%	0.1%	2469	75000
**BC309**			0.3%	839	75000
**BC341**	1.3%			3744	75000

Supplementary Table 1. Information on samples used for generating novel independent paired TCR dataset. All sorted cells in the positive subset were loaded in one lane and 17,000 cells from the negative subset were loaded in another lane. Both were processed using the 10x Chromium pipeline. Percentages are % of total CD8 T cells
